# Importance of Short-Term Neointimal Coverage of Drug-Eluting Stents in the Duration of Dual Antiplatelet Therapy

**DOI:** 10.3390/jcm13061730

**Published:** 2024-03-17

**Authors:** Joanna Fluder-Wlodarczyk, Sławomir Pawłowski, Piotr J. Chuchra, Tomasz Pawłowski, Wojciech Wojakowski, Pawel Gasior

**Affiliations:** 1 Division of Cardiology and Structural Heart Diseases, Medical University of Silesia in Katowice, 40-635 Katowice, Poland; joanna.fluder90@gmail.com (J.F.-W.); piotr.chuchra456@gmail.com (P.J.C.); wojtek.wojakowski@gmail.com (W.W.); 2 Students’ Scientific Society, Department of Cardiology, Faculty of Medical Sciences in Katowice, Medical University of Silesia, 40-635 Katowice, Poland; slawekkpawlowski@gmail.com

**Keywords:** drug-eluting stent, antiplatelet therapy, OCT

## Abstract

Dual antiplatelet therapy (DAPT) is mandatory after percutaneous coronary intervention (PCI) with drug-eluting stent (DES) implantation, but optimal DAPT duration remains a topic of intense discussion. The shorter regimen of DAPT might be especially beneficial for high-bleeding-risk (HBR) patients. Novel stent platforms have been designed with innovations that should facilitate vessel healing following stent implantation and enable short DAPT. This review aimed to summarize evidence of the neointimal coverage of short-term stents and their implications for DAPT duration. Results from histological and optical coherence tomography (OCT) studies confirm the significant role of uncovered struts after the implantation of a stent in patients presenting with late stent thrombosis. Several studies have shown favorable vessel healing at one month (with 83.2% of covered struts, on average) and three months following stent implantation (with 93.3% of covered struts, on average). Solely HBR patient trials have proven that one month of DAPT can be applicable and safe in this population. Three-month DAPT was tested in a more diverse population and remains effective and safe in comparison to a longer DAPT regimen. That evidence proves that short-term DAPT might be applicable, especially for HBR patients.

## 1. Introduction

Percutaneous coronary intervention (PCI) with drug-eluting stent (DES) implantation, followed by mandatory dual antiplatelet therapy (DAPT), is currently a standard treatment for patients with symptomatic coronary artery disease. Considering the high number of procedures performed worldwide, safety and efficacy are crucial. The goal of DAPT after DES implantation is to prevent stent thrombosis (ST) and, potentially, new plaque rupture in other segments of coronary arteries [1,2]. The latest recommendations of the European Society of Cardiology (ESC) recommend twelve months of DAPT as the default strategy (preferably aspirin with prasugrel or ticagrelor) for patients presenting with acute coronary syndrome (ACS) and six months of DAPT (preferably aspirin with clopidogrel) in patients with chronic coronary syndromes (CCSs). Alternatively, shortening of DAPT duration might be allowed. One month of DAPT might be considered for high-bleeding-risk (HBR) patients, followed by aspirin or P2Y12 inhibitor. Three to six months of DAPT should be applicable for event-free patients who are also not at high ischemic risk, preferably followed by a P2Y12 inhibitor [3,4]. However, due to constantly evolving stent technology, intravascular imaging optimization of stent implantation, and the introduction of new P2Y12 inhibitors, optimal DAPT duration remains a topic of intense discussion.

Around 40% of individuals undergoing PCI in routine clinical practice are considered HBR patients [5,6]. In definition, HBR patients assessed at the time of PCI have a risk of either ≥4% for Bleeding Academic Research Consortium (BARC) bleeding types 3 and 5 or ≥1% risk of intracranial hemorrhage at one year [7]. In this population, long-term DAPT (12 or 24 months) has not provided ischemic or mortality benefits, regardless of the coronary artery disease presentation and the complexity of the intervention. Also, extended DAPT was associated with an excess of bleeding complications [8]. Additionally, in populations treated with PCI, mortality associated with bleeding complications seems to be similar to myocardial infarction (MI)-related mortality [9,10]. In a meta-analysis of randomized trials, short DAPT duration (≤6–12 months) was associated with lower bleeding risk, with no apparent increase in ischemic complications compared to a prolonged strategy (≥12 months) [1,11].

Furthermore, second-generation DESs were designed with several improvements, including more biocompatible polymer coatings, better antiproliferative drugs, and thinner struts made of metal alloys. A new generation of DESs coated with bioresorbable polymer (BP) and polymer-free (PF) platforms have also been introduced. These innovations were designed to improve clinical outcomes, mainly by reducing thrombotic complications [12,13,14,15]. First-generation DESs were associated with a delayed endothelialization process, which translated into increased ST rates [16]. In theory, recent advances in DES technology should also facilitate vessel healing following stent implantation.

This review summarizes evidence of short-term neointimal coverage following DES implantation and its implications for DAPT duration.

## 2. Materials and Methods

To identify all relevant clinical trials and studies regarding the topic of this review, we undertook a literature search of PubMed (between 2002–2023) with the following keywords: “neointimal coverage or arterial healing and OCT and one month”, “neointimal coverage or arterial healing and OCT and three months”, “short DAPT”, “one month DAPT”, “three months DAPT”, “shortening of DAPT”, “Stent thrombosis and DES”, and “autopsy and DES”. We excluded studies in languages other than English. Reports with relevant titles and abstracts were retrieved for a full-text review. Primarily, we were interested in randomized clinical trials concerning the shortening of DAPT to one or three months, and we included all of these studies. Because this review aimed to present the importance of the short-term coverage of stents, we searched for OCT studies presenting the healing of platforms used in included trials. Overall, 79 studies were identified as relevant and included in this review.

## 3. Histological Studies of Healing Patterns in Different Stent Types

Several studies aimed to assess autopsies of patients who died following stent implantation to provide insights into arterial healing patterns. An autopsy registry of 40 individuals who previously underwent PCI compared 23 patients with first-generation DESs (Cypher and Taxus) vs. 25 patients with bare metal stents (BMSs) matched for age, sex, stented artery, and time following stent implantation. Among DES patients, 14 suffered from ST, 2 showed evidence of in-stent restenosis and died of MI or sudden cardiac death (CD), and 7 had patent stents and died of non-stent-related complications. The DES group demonstrated a significantly lower percentage of strut endothelialization (55.8 ± 26.5% vs. 89.8 ± 20.9%), significantly higher fibrin scores (2.3 ± 1.1 vs. 0.9 ± 0.8), and a higher percentage of struts surrounded by fibrin (49.3 ± 30.8% vs. 22.3 ± 17.8%) than the BMS group. Furthermore, a comparison of autopsy studies of DESs with late stent thrombosis (LST) and patent DESs revealed significantly more pronounced signs of delayed healing in the thrombosed DESs [16]. These results are consistent with the observation from another registry, which attempted to establish the major pathological risk factors for LST. The mentioned registry included autopsies of patients who died ≥30 days following first-generation DES implantation and compared 28 lesions with thrombus with 34 lesions without the presence of thrombus. Patent DES lesions demonstrated a higher percentage of endothelialization, greater neointimal thickness, and lower fibrin scores when compared to thrombosed lesions. This observation indicates that incomplete neointimal coverage is the most important histological predictor of LST, and the ratio of uncovered to total struts per section of >30% was identified as a predictor of ST [17]. Moreover, patients presenting with MI demonstrated delayed endothelialization compared with patients with stable angina following implantation of first-generation DESs [18].

The introduction of the new generation of DESs was associated with a reduction in ST rates [13]. Several pathological studies have sought to compare vascular response after implantation of second-generation everolimus-eluting stents (EESs) and first-generation DESs. EESs demonstrated significantly lower rates of uncovered struts, less fibrin deposition, and lower inflammation. However, there was no difference in the observed frequency of neoatherosclerosis [19,20]. Stent fractures in EESs were less frequent when compared to first-generation Cypher stents but were comparable to first-generation Taxus stents. Moreover, EESs presented greater stent coverage, irrespective of indications for angioplasty (stable coronary artery disease versus acute coronary syndrome) [20]. Additionally, a recent report aimed to evaluate early response to abluminal BP DESs compared to durable polymer (DP) DESs in human autopsy cases (implants with a duration of <90 days, with a median of 20 days for DP DESs and 17 days for BP DESs). The mentioned report demonstrated that focal, single-layer endothelial coverage occurred five days after implantation of both platforms, with low inflammation and a similar degree of fibrin deposition. Additionally, BP DESs showed greater strut coverage with smooth muscle cell infiltration when compared to DP DESs; however, both devices demonstrated suboptimal vessel healing at 30 days [21].

## 4. Neointimal Coverage Assessed by OCT

**Figure 1:**
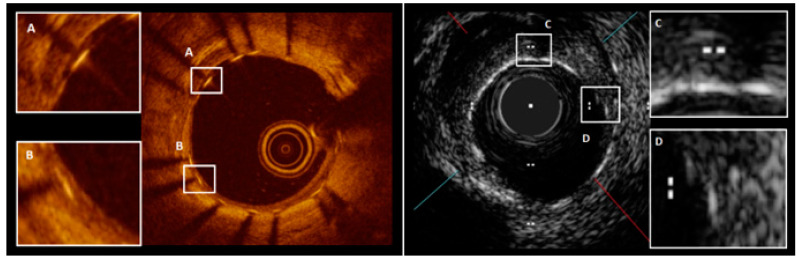
The left side presents a frame from an OCT examination one month following stent implantation with either uncovered struts (**A**) or covered struts (**B**). The right side shows a vessel one month following stent implantation visualized by HD-IVUS. The frame comprises uncovered (**C**) and covered (**D**) struts. Both pictures were obtained from procedures performed within the Division of Cardiology and Structural Heart Diseases, Medical University of Silesia in Katowice.

Histological assessment of different DES types has significantly expanded current knowledge on vessel healing following PCI. However, the adoption of this method is restricted due to limited sample availability. In vivo assessment of neointimal coverage following DES implantation seems more suitable for accurately defining the safety profile. Optical coherence tomography (OCT) is an intravascular imaging technique based on near-infrared light emission. This modality is characterized by a high resolution (15–20 µm axial and 20–40 µm lateral). Therefore, OCT enables precise quantification and detection of tissue coverage, which might be missed by other imaging modalities, even by high-definition intravascular ultrasound (HD-IVUS) [22,23,24] (Figure 1). Several preclinical studies have investigated the accuracy of OCT and proven its reliability. Those studies have shown good agreement between OCT and histological evaluations of neointimal formation following stent implantation [25,26,27]. However, it is important to emphasize that we cannot distinguish the composition of tissue on the strut surface. A strut is considered to be covered by histology when luminal endothelial cells with two abluminal layers of smooth muscle cells and matrix are present. A recent ex vivo human autopsy study sought to evaluate the accuracy of neointimal coverage assessment by OCT and compare it to histology evaluation. The mentioned study established that the most precise cut-off value for identifying complete strut coverage is a neointimal thickness of ≥40 µm. This threshold provided high specificity (91.0%) and sensitivity (99.3%), with excellent positive and negative predictive values (98.6% and 95.6%, respectively). In contrast, qualitative OCT strut evaluation without any neointimal thickness cut-off showed poor specificity (37.5%) but good sensitivity (100%) [28]. Moreover, qualitative assessment of strut coverage is characterized by good intra- and inter-observer reproducibility [29,30].

### Optical Coherence Tomography (OCT) and High-Definition Intravascular Ultrasound (HD-IVUS)

OCT studies also confirmed the significant role of uncovered stent struts in patients presenting with LST. In a study that included 54 patients after DES implantation, 18 presented with LST and generally had a higher percentage of uncovered stent struts (12.27% vs. 4.14%). Also, 72% of patients with LST presented segments with >30% uncovered struts. Nonetheless, LTS is not always caused by the absence of neointimal coverage. The mechanism of LTS in three individuals was likely due to neointimal hyperplasia or in-stent or peri-stent plaque rapture [31]. In another study, the frequency of uncovered struts was assessed between 6 and 18 months after DES implantation. A cut-off of ≥5.9% was established as a predictor of major events, such as cardiovascular death, MI, and ST [32].

Those qualities prove OCT as the main modality in safety and efficacy assessment of various stent platforms, both in experimental and clinical settings.

## 5. Assessment of Stent Healing by OCT One Month following PCI and Safety of One-Month DAPT

OCT studies demonstrated favorable healing patterns of novel stent platforms at early stages following implantation, which, in theory, should enable safe discontinuation of DAPT. Several studies on different platforms were conducted to investigate strut coverage one month following stent implantation. These studies provided a foundation for clinical trials evaluating the safety of one-month DAPT following PCI.

Until recently, HBR patients were frequently treated with BMS, despite data confirming the long-term superiority of DES, because it was the only platform enabling one-month DAPT due to rapid vessel healing. A similar healing pattern was achieved by the Biofreedom (Biosensors Europe SA, Morges, Switzerland) polymer- and carrier-free drug-coated stent (DCS), with ≈90% of the Biolimus A9 released from the surface within 48 h of implantation [33]. OCT studies have confirmed rapid vessel healing following DCS implantation. In the EGO-BIOFREEDOM study, at one-month follow-up, 85.8% of stent struts were covered with tissue [34]. Furthermore, another OCT study in the STEMI population compared the difference in vascular healing between patients with ruptured plaques (RPs) and non-ruptured plaques (NRPs) one month following PCI with DCS implantation. Interestingly, the rate of uncovered struts was similar between the two groups, with 26.5% in the RP group and 28.1% in the NRP group [35]. The LEADERS FREE trial compared the safety and efficacy of one-month DAPT after PCI with DCS and BMS solely in HBR patients. [36]. In this trial, the superiority of DCS was confirmed with respect to the primary safety endpoint (composite of CD, MI, or ST occurred in 9.4% in DCS vs. 12.9% in the BMS group) and to the primary efficacy endpoint (clinically driven target lesion revascularization 5.1% in DCS vs. 9.8% in the BMS group) at one-year follow-up [37], which was sustained up to the two-year follow-up [38]. The LEADERS FREE II trial was designed to reproduce the results of the LEADERS FREE trial in a predominantly North American cohort of HBR patients treated with DCS and as a pivotal trial for Food and Drug Administration (FDA) approval of the BioFreedom stent. Its results supported the safety and efficacy if a DCS followed by one-month DAPT regimen in the HBR population when compared to BMS [39]. Furthermore, new polymer-free Biolimus-A9 coated thin-strut (84–88 µm) cobalt-chromium stents (Co-Cr DCS) were investigated in an HBR population with one-month DAPT following PCI in the LEADERS FREE III trial. The new Co-Cr DCSs were confirmed to be as safe as DCSs and superior to a BMS in terms of efficacy in the propensity-matched cohort of patients from the LEADERS FREE trial at one-year follow-up [40].

The next platform evaluated for a short DAPT regimen was a zotarolimus-eluting stent (ZES, Resolute Onyx, Medtronic, Santa Rosa, CA, USA) with a biocompatible, durable BioLinx™ polymer. It has thin 81 µm struts made of a cobalt alloy outer shell and a platinum-iridium inner core [41]. A preclinical study showed superior thromboresistance and parallel healing of the Resolute Onyx platform compared to a polymer-free Biolimus-eluting stent [42]. In an OCT study in humans, Resolute Onyx stents showed favorable vessel healing, with 88% covered struts and 92.3% of the total lumen surface area with complete stent coverage at one-month follow-up. Most of the included patients (86.7%) presented with unstable angina [43]. Furthermore, a single-center, retrospective registry analysis aimed to evaluate the 1-year clinical outcomes of patients treated with ZESs in a real-world population, including patients with ultra-high-risk characteristics. In this study, the ZESs showed favorable 1-year clinical outcomes, with a low rate of target lesion failure, cardiovascular deaths, target lesion revascularization, and ST [44]. These studies provided a foundation for the Onyx One Global and Onyx One Clear trials. The Onyx One Global trial included HBR patients undergoing PCI who were randomized to receive either a DP ZES or DCS with one-month DAPT after the index procedure [45]. At one-year follow-up, ZESs proved noninferiority to DCSs [46]. Also, the final 2-year results of the Onyx One trial showed comparable safety and effectiveness between the two platforms [47]. These data were supported by the ONYX Clear study [48].

Several clinical trials have aimed to evaluate safety of one-month DAPT following implantation of a durable polymer-coated everolimus-eluting stent (DP-EES, Xience, Abbott Vascular, Santa Clare, CA, USA) [49,50,51]. OCT assessment of early healing after EES implantation demonstrated 80.26 ± 16.43% covered struts at one-month follow-up [52]. In the STOP DAPT 2 study, after EES stent implantation, patients were randomized to either one-month DAPT followed by clopidogrel monotherapy or 12-month DAPT. The study confirmed both the noninferiority and superiority criteria for shorter DAPT [49]. However, in the STOP DAPT 2 ACS, among patients presenting with ACS, the short DAPT strategy was associated with an increase in cardiovascular events but a reduction in bleeding events and failed to meet the criteria for noninferiority in comparison to the 12-month DAPT strategy [50]. Also, in the Xience 28 study, one-month DAPT was associated with a lower rate of severe bleeding and no increase in ischemic events in the HBR population compared to six-month DAPT [51].

**Figure 2:**
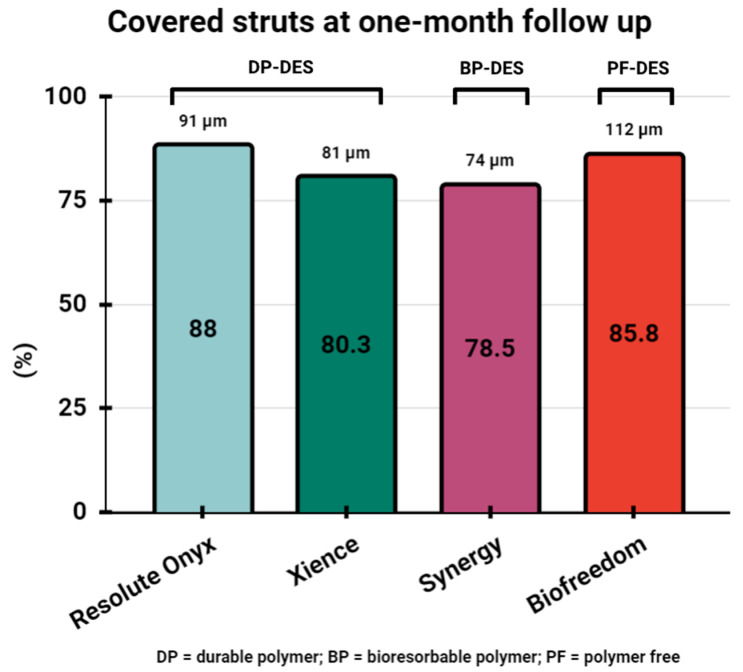
Covered struts at one-month follow-up in tested DES platforms.

Another EES but with a abluminal biodegradable polymer coating (BP-EES, Synergy, Boston Scientific, Marlborough, MA, USA) was also perceived as a platform providing favorable early arterial healing, with 82.4 ± 12.4% of struts covered in an OCT study two weeks following implantation. Also, OCT analysis comparing ACS and non-ACS patients showed that the frequency of covered struts was similar between the two groups (83.5 ± 12.5% and 80.6 ± 12.5%, respectively) [53]. Furthermore, a study in STEMI patients treated with either BP-EESs or DP-EESs demonstrated that two weeks following implantation, the proportion of covered struts in the BP-EES group (42.4 ± 15.4%) was significantly higher than in the DP-EES group (26.3 ± 10.1%, *p* < 0.001) [54]. Another OCT study in non-ST segment elevation ACS (NSTE-ACS) patients one month after PCI demonstrated mean coverage of 78.5 ± 10%, with 79.2% of patients having at least 70% of their struts covered [55]. This favorable healing pattern of BP-EESs was tested in the POEM trial, which aimed to evaluate the safety of PCI with a BP-EES followed by one-month DAPT in HBR patients in the Italian population. At one-year follow-up, the primary outcome (prevalence of CD, MI, probable/definite ST) had occurred in 4.82% of patients, demonstrating noninferiority compared with the predefined objective performance criterion; however, it needs to be stressed that the study was prematurely terminated due to slow enrollment [56]. Also, in the SENIOR trial, a short DAPT regimen (one month for CCS and six months for ACS) in patients older than 75 years was tested between patients treated with BMSs and BP-EESs. The occurrence of all-cause mortality, MI, stroke, and ischemia-driven target lesion revascularization was lower in the BP-EES compared to the BMS group [57]. The one-month strut coverage of the abovementioned platforms is summarized in Figure 2.

## 6. Assessment of Stent Healing by OCT Three Months following PCI and Safety of Three-Month DAPT

**Figure 3:**
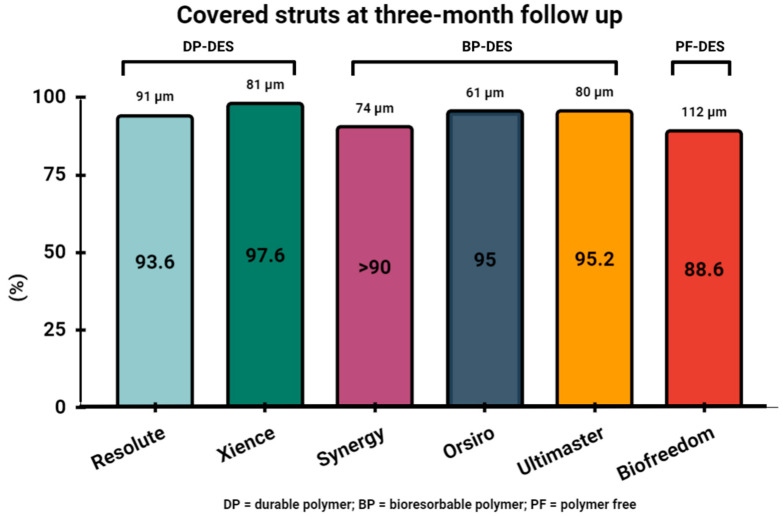
Covered struts at 3-month follow-up in tested DES platforms.

While one-month DAPT seems appropriate mostly for selected HBR populations, three months of DAPT might apply to a wider range of patients. Numerous OCT studies have demonstrated favorable stent coverage at a three-month follow-up in different stent platforms. In the EGO-BIOFREEDOM study, DCSs demonstrated strut coverage of 88.6% at three-month follow-up [34]. Also, a recent report revealed nearly completed early vascular healing of both DCS and EES platforms, with a significantly higher frequency of covered struts in the DP_EES group (97.6% vs. 94.7%; *p* < 0.001) [58]. Furthermore, almost complete coverage at three-month follow-up was confirmed in the ZES platform, with 93.6% of struts covered [59]. Moreover, BP-coated sirolimus-eluting stents (SESs) (Orsiro, Biotronik AG, Bulach, Switzerland) and ultrathin struts (60 µm for 2.25 to 3.0 mm and 80 µm for >3.0 mm sizes) demonstrated coverage of >95% in several studies at three-month follow-up [60,61,62]. The percentage of covered struts was also high for other biodegradable polymer-coated platforms, including BP-EESs (>90%) [63,64,65] and BP-coated sirolimus eluting stents (BP-SESs, Ultimaster, Terumo, Tokyo, Japan) (95.2%) [66]. Direct comparison of vascular healing after DP-DES and BP-DES implantation revealed comparable coverage at three-month follow-up yet demonstrated different neointimal characteristics in light property analysis. DP-DESs were associated with higher light intensity and higher light attenuation/backscatter values than BP-DESs, which might indicate a favorable vascular healing process following BP-DES implantation [67]. Also, quantitative OCT analysis of neointimal quality and coverage, likewise, indicated comparable neointimal characteristics three months following PCI in the ACS vs. CCS setting [68]. Furthermore, implantation technique matters, since OCT-guided PCI improved vascular healing at three-month follow-up compared with angiography-guided DES implantation [62,69]. A summary of the three-month strut coverage of the mentioned platforms is presented in Figure 3.

The role of OCT in the early cessation of DAPT was investigated in the DECECT-OCT trial. Arterial healing was assessed three months following stent implantation. and patients with ≤6% uncovered struts were assigned to three-month DAPT. The ischemic and bleeding event rates were low and comparable with those of the twelve-month DAPT groups [69]. The RESET trial proved the safety of three months of DAPT after Endeavor ZES (Medtronic, Santa Rosa, CA, USA) implantation vs. twelve months of DAPT, with no significant differences in ischemic and bleeding events at one-year follow-up. However, it must be emphasized that patients with very high risk were excluded [70]. The STOPDAPT trial tested the safety of short DAPT after implantation of a DP-EES. Patients were enrolled if the physician’s opinion was that three months of DAPT was suitable and they had not undergone previous PCI with stents other than DP-EESs. Outcomes were compared with the historical control arm, which was constituted from the DP-EES group from the RESET trial, and no significant differences were found between the studied groups [71]. The SMART CHOICE trial compared P2Y12 inhibitor monotherapy after three months of DAPT with twelve months of DAPT in patients undergoing PCI with newer-generation DESs. The tested regimen proved noninferiority regarding the incidence of major adverse cardiac and cerebrovascular events in the enrolled low-risk population [72].

Solely high-bleeding-risk patients were evaluated in the XIENCE Short DAPT and the EVOLVE Short DAPT trials. Patients were eligible for discontinuation of P2Y12 inhibitors after three months of event-free DAPT following the implantation of EESs and BP-EESs, respectively. In the EVOLVE Short DAPT trial, three months of DAPT followed by aspirin was associated with a favorable rate of ischemic events in selected patients (patients with acute myocardial infarction or complex lesions were excluded) [73]. In the XIENCE Short DAPT trial, three months of DAPT was also associated with lower rates of significant bleeding and a lower incidence of ST compared to twelve months of DAPT [51].

Patients with high ischemic risk who underwent successful PCI with BP-SES stent implantation due to ACS were eligible for the TICO trial, in which three months DAPT followed by ticagrelor monotherapy was compared with twelve months of DAPT with ticagrelor. A shorter regimen resulted in a reduction in major bleeding and cardiovascular events at one-year follow-up [74]. Also, in the TWILIGHT trial in patients at high-risk for bleeding or ischemic events, monotherapy with ticagrelor after completing three months of DAPT compared to twelve months of ticagrelor-based DAPT was associated with a lower rate of clinically relevant bleeding, without an increase in the incidence of ischemic events [75].

Clinical trials concerning one-month and three-month DAPT are summarized in Table 1.

## 7. Future Perspectives

The use of OCT to assess arterial healing to guide DAPT duration after stent implantation is highly promising. However, to date, there are no guidelines on OCT-guided individualization of DAPT duration based on the coverage of stent struts, since this OCT parameter is not a consensus-validated surrogate marker for DAPT discontinuation. However, the evaluation of stent healing using OCT is time-consuming, making this method challenging to apply in daily practice. Fortunately, this limitation might be overcome by automatizing this process. In recent years, a few algorithms for automated evaluation of stent strut coverage have been introduced [76,77,78,79], but to the best of our knowledge, these methods are not available commercially yet. Furthermore, due to the relatively low incidence of ST in new-generation DESs, large-sample-size randomized trials are required to demonstrate potential advantages of OCT-guided antiplatelet therapy.

## 8. Conclusions

OCT is an applicable tool in evaluating the safety and efficacy of stent platforms. Studies have shown favorable vessel healing after PCI procedures with newer DES implantation at one-month and three-month follow-up. Short-term DAPT after the implantation of novel stents is associated with a reduction in bleeding events without increased risk of ischemic events. This regimen might be especially beneficial for HBR patients.

## Figures and Tables

**Table 1:** Summary of trials presented in the review.

Trial	Design	Stent Type	P2Y12 Inhibitors	Endpoints	Outcomes	Duration of the Study
LEADERS FREE I [36,37,38]	1 M DAPT following DCS implantation vs. 1 M DAPT following BMS implantation, both followed by ASA	SS-DCS, BMS	Clopidogrel	Primary efficacy: clinically driven TLR at one-year follow-up Primary safety: composite of CD, MI, and ST at one-year follow-up Secondary: bleeding events	The use of DCS followed by 1 M DAPT in HBR patients is superior to BMS in regard to safety and efficacy endpoints.	2012–2015
LEADERS FREE II [39]	1 M DAPT following DCS implantation vs. 1 M DAPT following BMS implantation (BMS group from LF I trial), both followed by aspirin	SS-DCS, BMS	Clopidogrel	Primary efficacy: clinically indicated TLR at one-year follow-up Primary safety: composite of MI and CD at one-year follow-up Secondary: composite of CD, MI, or ST	The use of DCS followed by 1 M DAPT in HBR patients is superior to BMS in regard to safety and efficacy endpoints.	2017–2018
LEADERS FREE III [40]	1 M DAPT following CoCr-DCS implantation vs. 1 M DAPT following BMS implantation and 1 M DAPT following CoCr-DCS implantation vs. 1 M DAPT following DCS implantation (BMS and DCS group taken from LF I trial) followed by aspirin	CoCr-DCS, BMS, SS-DCS	Clopidogrel	Primary efficacy: clinically indicated TLR Primary safety: composite of CD, MI, and definite/probable ST	CoCr-DCS proved noninferior to the SS-DCS for safety and superior to the BMS for efficacy in HBR patients treated with 1 M of DAPT.	2017–2019
ONYX ONE Global [45,46,47]	1 M DAPT following ZES implantation vs. 1 M DAPT following DCS implantation, both followed by long-term aspirin or clopidogrel alone	ZES, DCS	Clopidogrel, prasugrel, ticagrelor	Primary: composite of CD, MI, or ST at 1-year follow-up Secondary: target lesion failure at 1 year	ZES proved to be noninferior to DCS in HBR patients with regard to primary and secondary endpoints at one-year follow-up. At 2-year follow-up, ZES and DCS had similar outcomes for the primary and secondary endpoints.	2017–2018
ONYX ONE CLEAR [48]	Multicenter, nonrandomized study evaluating the safety and effectiveness of 1 M DAPT followed by SAPT in patients who were event-free before DAPT discontinuation	ZES	Clopidogrel, prasugrel, ticagrelor	Primary: composite of CD or MI between 1 M and 1 year Secondary: rates of all-cause death, CD, major adverse cardiac events, target vessel failure, target lesion failure, any coronary revascularization procedure, definite/probable ST, stroke, and bleeding	Favorable safety and effectiveness between 1 M and 1 year were demonstrated among HBR patients unable to undergo prolonged DAPT therapy who were treated with Onyx Resolute ZES.	2018–2020
STOPDAPT-2 [49]	1 M DAPT followed by clopidogrel monotherapy vs. 12 M DAPT	CoCr-EES	Clopidogrel, prasugrel	Primary: composite of cardiovascular death, MI, definite ST, ischemic or hemorrhagic stroke, or major or minor bleeding at 12 M follow-up Secondary: a composite of cardiovascular death, MI, definite ST, ischemic or hemorrhagic stroke, and the bleeding endpoint of major or minor bleeding assessed at 12 M follow-up	1 M of DAPT followed by clopidogrel monotherapy was noninferior and superior to 12 M of DAPT.	2015–2018
STOPDAPT 2 ACS [50]	1–2 M DAPT followed by clopidogrel monotherapy vs. 12 M DAPT in ACS patients	CoCr-EES	Clopidogrel, prasugrel	Primary: composite of cardiovascular death, MI, definite ST, ischemic or hemorrhagic stroke, or major or minor bleeding at 12 M follow-up Secondary: a composite of cardiovascular death, MI, definite ST, ischemic or hemorrhagic stroke, and the bleeding endpoint of major or minor bleeding assessed at 12 M follow-up	In ACS patients, clopidogrel monotherapy after 1 to 2 M of DAPT failed to prove noninferiority to 12 M DAPT, with an increase in cardiovascular events (2.76% vs. 1.86%), despite a lower occurrence of major bleeding events (0.54% vs. 1.17%) at 12 M follow-up.	2018–2021
POEM [56]	1 M DAPT followed by ASA or OAC monotherapy following Synergy PtCr-EES implementation vs. OPC	PtCr-EES	Clopidogrel, prasugrel, ticagrelor	Primary: composite of CD, MI, or definite or probable ST at 12 M Secondary: all-cause death, CD, MI, ST, target vessel revascularization, TLR, major bleeding according to BARC criteria, cerebrovascular events, and target lesion failure	1 M DAPT followed by ASA or OAC monotherapy was noninferior to OPC based on the occurrence of the primary endpoint (4.82% vs. 9.4% with a noninferiority margin of 3.85%) at one year follow-up.	2017–2020
SENIOR [57]	CCS: EES implantation followed by 1 M DAPT vs. BMS implantation followed by 1 M DAPT ACS: EES implantation followed by 6 M DAPT vs. BMS implantation followed by 6 M DAPT	EES, BMS	Clopidogrel, prasugrel, ticagrelor	Primary: composite of all-cause mortality, MI, stroke, or IDTLR at 1-year follow-up Secondary: bleeding complications (BARC 2–5 and BARC 3–5); definite or probable ST, all revascularisations; all components of the primary endpoint; and cardiovascular death, at 30 days, 180 days, 365 days, and 2 years	EES implantation followed by short-duration DAPT resulted in a lower occurrence of the primary endpoint than BMS implantation followed by short-duration DAPT (12% vs. 16%) at 1-year follow-up, proving to be better for people 75 years or older.	2014–2017
DETECT-OCT [69]	OCT-guided PCI vs. angiographically guided PCI, according to the percentage of uncovered struts: 3 M DAPT followed by ASA (<6% uncovered struts at 3 M follow-up) vs. 12 M DAPT (>6% uncovered struts at 3 M follow-up)	EES, BES	Clopidogrel	Primary: differences in 3 M coverage between EES vs. BES and OCT-guided and angiographic PCI Secondary: a composite of CD, MI, definite or probable ST, and major bleeding during 12 M follow-up	− Similar strut coverage between EES and BES, and OCT-guided PCI group had a lower percentage of uncovered stents than the angiographic PCI group (7.5% vs. 9.9%, respectively) at 3 M follow-up. − During 12 M follow-up, the composite event occurred in 0.3% in the 3 M DAPT group and 0.2% in 12 month DAPT group.	2013–2017
RESET [70]	3 M DAPT followed by ASA monotherapy following E-ZES implantation vs. 12 M DAPT standard therapy	ZES, EES, SES	Clopidogrel	Composite of death from cardiovascular causes, MI, ST, IDTR, or bleeding at 1-year follow-up	3 M DAPT following E-ZES implantation was noninferior to standard therapy.	2009–2012
STOPDAPT [71]	3 M DAPT followed by ASA vs. 12 M DAPT in historical group (the CoCr-EES group in the RESET trial)	CO-Cr EES	Clopidogrel	Primary: Composite of cardiovascular death, MI, stroke, definite ST, and major/minor bleeding at 1 year Secondary: death, MI, stroke, possible/probable/definite ST, bleeding, TLR, TVR, coronary artery bypass grafting, and any coronary revascularization	3 M DAPT was at least as safe as 12 M DAPT.	2012–2014
SMART-CHOICE [72]	3 M DAPT followed by P2Y12 inhibitor vs. 12 M DAPT	EES, ZES, SES	Clopidogrel, prasugrel, ticagrelor	Primary: composite of all-cause death, MI, or stroke at 12 M following PCI Secondary: the components of the primary endpoint; CD; TLR; TVR; any revascularization; ST; BARC bleeding type of at least 2 or 3; a composite of death, MI, cerebrovascular events, or any revascularization at 12 M follow-up’ and each component of primary and secondary endpoints at 2 and 3 years.	3 M DAPT followed by P2Y12 inhibitor monotherapy was noninferior to 12 M DAPT in regard to the occurrence of major adverse cardiac and cerebrovascular events.	2014–2018
EVOLVE Short DAPT [73]	3 M DAPT followed by ASA (event-free patients at 3 M follow-up) vs. 12 M DAPT	EES	Clopidogrel, prasugrel or ticagrelor	Primary: all-cause death/MI and study stent-related definite/probable ST Secondary: the rate of bleeding (types 2, 3, and 5 according to BARC) in patients not receiving chronic anticoagulation	3 M DAPT was associated with favorable rates of ischemic events in HBR patients.	2016–2019
XIENCE Short DAPT [51]	XIENCE 28: 1 M DAPT followed by ASA vs. 6 M DAPT XIENCE 90: 3 M DAPT followed by ASA vs. 12 M DAPT	EES	Clopidogrel, prasugrel or ticagrelor	Primary: the composite of all-cause death or MI Secondary: Bleeding types 2–5 according to BARC and definite or probable stent thrombosis	1–3 M DAPT in HBR patients was noninferior in ischemic outcomes, and the incidence of ST was low, which may be associated with a lower incidence of major bleeding.	2017–2020
TICO [74]	3 M DAPT followed by ticagrelor vs. 12 M DAPT in ACS patients	BP-SES	Ticagrelor	Primary: a composite of major bleeding and adverse cardiac and cerebrovascular events within 1 year Secondary: major or minor bleeding, death, MI, ST, stroke, and TVR	3 M DAPT was associated with a statistically significant reduction in the composite outcome of major bleeding and cardiovascular events at 1 year.	2015–2023
TWILIGHT [75]	3 M DAPT followed by ticagrelor vs. 12 M DAPT	DES	Ticagrelor	Primary: bleeding types 2, 3, and 5 according to BARC Secondary: death from any cause, nonfatal MI, or nonfatal stroke	3 M DAPT followed by ticagrelor resulted in reduction in rates of bleeding, without an increase in ischemic events.	2015–2019

M—month, DAPT—dual antiplatelet therapy, DCS—drug-coated stent, BMS—bare-metal stents, ASA—aspirin, SS-DCS—stainless-steel drug-coated stent, TLR—target lesion revascularization, CD—cardiac death, MI—myocardial infarction, ST—stent thrombosis, HBR—high bleeding risk, LF I—LEADERS FREE I, CoCr-DCS—cobalt-chromium drug-coated stent, ZES—zotarolimus-eluting stent, SAPT—single antiplatelet therapy, CoCr-EES—cobalt-chromium everolimus-eluting stent, ACS—acute coronary syndrome, OAC—oral anticoagulant, PtCr-EES—platinum-chromium everolimus-eluting stent, OPC—objective performance criterion, BARC—Bleeding Academic Research Consortium, CCS—chronic coronary syndrome, IDTLR—ischemia-driven target lesion revascularization, OCT—optical coherence tomography, PCI—percutaneous coronary intervention, BES—Biolimus-eluting stent, E-ZES—Endeavor zotarolimus-eluting stent, SES—sirolimus-eluting stent, TVR—target vessel revascularization, BP-SES—bioresorbable polymer sirolimus-eluting stent, DES—drug-eluting stent.

## Data Availability

Not applicable.
